# MiR-519d facilitates the progression and metastasis of cervical cancer through direct targeting Smad7

**DOI:** 10.1186/s12935-016-0298-1

**Published:** 2016-03-22

**Authors:** Jue-Yu Zhou, Si-Rong Zheng, Jie Liu, Rong Shi, Hai-Lang Yu, Min Wei

**Affiliations:** Institute of Genetic Engineering, School of Basic Medical Sciences, Southern Medical University, Guangzhou, 510515 Guangdong People’s Republic of China; Department of Obstetrics and Gynecology, Nanfang Hospital, Southern Medical University, Guangzhou, 510515 Guangdong People’s Republic of China

**Keywords:** MiR-519d, Cervical cancer, Metastasis, Smad7, Proliferation

## Abstract

**Background:**

MicroRNAs (miRNAs) play pivotal roles in the development of various cancer types, including cervical cancer.

**Methods and results:**

In this study, we showed that miR-519d, a miRNA within the chromosome 19 miRNA cluster, was significantly upregulated in cervical cancer tissues, compared with non-tumorous cervical samples. Suppression of miR-519d markedly attenuated the migration and invasion of HeLa and SiHa cervical cancer cells. Additionally, miR-519d inhibited the apoptosis of cervical cancer cells, and the proliferation of cervical cancer cells was also affected following transfection of miR-519d inhibitor. Moreover, we identified Smad7 to be a novel target of miR-519d in cervical cancer cells. MiR-519d matched the 3′-UTR of Smad7 mRNA. Transfection with miR-519d mimics led to apparent downregulation of Smad7 both at the mRNA and protein levels. Luciferase reporter analysis revealed that miR-519d reduced the luciferase activity of Smad7 mRNA 3′-UTR through matching site-dependent manner. And more notably, suppression of Smad7 remarkably restored the migration and invasion of miR-519d-depleted cervical cancer cells.

**Conclusion:**

Taken together, these findings implicated that miR-519d promoted the progression and metastasis of cervical cancer through targeting Smad7.

## Background

Cervical cancer represents the third most frequent cancer and the fourth leading cause of cancer-related morality in women worldwide. According to recent global cancer statistics, it is estimated that there are approximate 530,000 new cervical cancer cases annually, and 275,000 cervical cancer-related deaths [1]. In addition, the prognosis of cervical cancer remains unsatisfactory, with a 5 year overall survival of approximate 36.5 % in United States [2]. Therefore, cervical cancer remains a significant threat to female health. Mounting studies indicated that distant metastasis is a key determinant that predicts the survival of cervical cancer patients. Patients with metastatic cervical cancer (mCC) have markedly worsened prognosis, compared with non-mCC patients [3]. However, the molecular mechanism underlying cervical cancer invasion and metastasis remains largely unclear, limiting the therapeutic options in the management of this deadly disease.

Cervical cancer metastasis involves complex molecular mechanisms. A variety of signaling pathways, including TGF-β/Smads, Wnt/β-catenin and JAK/STATs pathways, have been implicated in the regulation of cervical cancer metastasis and invasion. Serving as a master regulator of cell motility and migration, TGF-β/Smads signaling has been extensively documented to play a pivotal role in determining the metastasis of various cancer types, including cervical cancer. It was reported that hyperactivation of TGF-β signaling was associated with lymph node metastasis in cervical cancer [4, 5]. TGF-β pathway mainly signals through Smad proteins. The TGF-β receptors directly phosphorylate receptor-activated Smads (R-Smads) to trigger their nuclear translocation. Subsequently, nuclear translocated R-Smads interact with Smad4 to form Smad complex and direct the transcription of target genes through their transcription factor activities. In addition to R-Smads and Smad4, Smad6 and Smad7 serve as inhibitory Smads (I-Smads) to attenuate the activity of TGF-β signaling. Studies have indicated that overexpression of Smad7 impaired the metastasis and invasion of tumor cells via negative regulation of TGF-β [6, 7]. It was reported that Smad7 was rarely mutated in human cervical cancer tissues [8]. However, it remains unclear whether posttranscriptional regulation of Smad7 may contribute to the development of cervical cancer.

MicroRNAs (miRNAs) are a conserved class of 22 bp long non-coding RNAs that have been discovered over a decade ago. Since their discoveries, numerous studies have highlighted critical involvement of miRNAs in the regulation of gene expression and cellular signaling transduction [9]. miRNAs primarily regulate gene expression through directly binding to the matched sites of mRNAs and triggering rapid decay of target mRNAs. In this way, miRNAs play important roles in diverse biological processes, such as organism development, immune regulation and tumorigenesis [10, 11]. Currently, many miRNAs have been documented to possess oncogenic or tumor-suppressive properties. For example, miR-21 has been repeatedly reported to facilitate tumor progression through targeting assorted tumor-suppressors, such as Bcl-2, PDCD4 and PTEN [12–14]. MiR-519d triggers multiple gene targets, including p21, PTEN, AKT3 and TIMP2 to potentiate HCC development [15]. Xiaoxia Hu et al. reported that miR-200a and miR-9 might exert important regulatory roles in cervical cancer development and metastasis, and potentially served as prognostic indicators of cervical cancer patients’ survival. However, to date, the regulatory role of miRNAs in the development and metastasis of cervical cancer remains poorly documented [16].

Given the information above, we investigated the miRNAs that potentially regulates the development and metastasis of cervical cancer. We identified that miR-519d was highly expressed in cervical cancer specimens. In addition, we showed that miR-519d facilitated cervical cancer proliferation, migration and invasion in vitro. Moreover, we identified Smad7 to be a novel target gene of miR-519d. Depletion of Smad7 regained the migration and invasion of cervical cancer cells following miR-519d inhibition. Our findings implied that high expression of miR-519d may contribute to the development of cervical cancer through targeting Smad7, providing novel insight into the role of miRNAs in the regulation of cervical cancer metastasis.

## Methods

### Patients and samples

Cervical cancer tissues and adjacent normal tissues were obtained from 20 patients, who underwent cervical surgical resection without preoperative systemic therapy at Nanfang Hospital of Southern Medical University between September 2013 and January 2015. The major pathologic variables were obtained and recorded before surgical resection. After surgical removal, the tissues were immediately frozen using liquid nitrogen. All human tissues were collected in accordance with protocols approved by the Ethics Committee of the Nanfang Hospital of Southern Medical University.

### Cell culture, construct and transfection

HeLa and SiHa cells were obtained from Shanghai Institute of Cell Biology, Academic Sinica, and cultured in high-glucose DMEM (Invitrogen) supplemented with 10 % fetal bovine serum (Hyclone, South America) at 37 °C and 5 % CO_2_. Smad7 shRNA was obtained from Genechem company (Shanghai, China). The miR-519d mimics and inhibitor were obtained from Invitrogen (Carlsbad, CA). The transfection of miR-519d mimics, inhibitor and shSmad7 oligo was performed using Lipofectamine 2000 (Invitrogen, Carlsbad, CA) according to the manufacturer’s instruments.

### MiRNA extraction, reverse transcription and real-time PCR detection

The total RNA was extracted using Trizol reagent, and reversely transcribed into cDNA. Real-time PCR detection of miR-519d was conducted as reported by Francesca Fornari et al. [15]. The primers used for RT-PCR detection included: miR-519d, 5′-ACA CTC CAG CTG GGC AAA GTG CCT CCC T-3′, and 5′-CTC AAC TGG TGT CGT GGA-3′; U6, 5′-CTC GCT TCG GCA GCA CA-3′, and 5′-AAC GCT TCA CGA ATT TGC GT-3′; Smad7, 5′-CTC GGT GAA ACC CGT CCA T-3′, and 5′-GAG CAA ATC CTT TCC GAC CAG-3′; GAPDH, 5′-CGG AGT CAA CGG ATT TGG TCG TAT-3′ and 5′-AGC CTT CTC CAT GGT GGT GAA GAC-3′.

### Cell migration and invasion assay

For cell migration assay, 5 × 10^4^ HeLa or SiHa cells were placed into the upper part of transwell chamber containing a non-coated membrane in 200 μl serum-free DMEM medium. As to cell invasion assay, 5 × 10^4^ HeLa or SiHa cells were seeded into the upper chamber of coated with 40 μl of 2 mg/ml Matrigel (BD Matrigel™ matrix; BD Bioscience, Heidelberg, Germany). Six hundred micro-liters DMEM medium containing 20 % FBS was added into the lower part of the chamber. After incubation for 24 h, the membranes were stained using 2 % crystal violet for 15 min. The cells that traveled through the membranes were examined using a digital light microscope. Each experiment has been repeated at least three times.

### Western blot analysis

Cervical cancer samples and cells were homogenized using a lysis buffer containing 50 mM Tirs-Cl, pH 7.4, 120 mM NaCl, 1 % NP-40, 0.2 % SDS, 1 mM EDTA and complete protease inhibitor cocktail (Roche Diagnostics, Basel, Switzerland), and centrifuged for 20 min at 13,000*g*, 4 °C. The protein concentration of cell lysate was analyzed using BCA protein assay kit (Bio-Rad, Hercules, CA). The protein samples were separated by SDS-PAGE and transferred into PVDF membranes. The membranes were blocked using blocking solution (150 mM NaCl, 20 mM Tris, pH 8.0, 0.05 % Tween-20, 5 % non-fat milk). Thereafter, the membranes were incubated with the indicated primary antibodies: rabbit polyclonal anti-Smad7 antibody (Santa Cruz Biotechnology, Santa Cruz, CA) and mouse monoclonal anti-GAPDH antibody (TA-08, Zhongshan Golden Bridge, Beijing, China). Secondary antibody incubation was conducted using horseradish peroxidase (HRP)-conjugated goat anti-mouse and anti-rabbit antibodies. The protein bands were visualized using ECL methods according to the manufacturer’s instruments (Zhongshan Golden Bridge, Beijing, China). The densities of protein bands were relatively quantified using ImageJ software (WS Rasband, ImageJ, NIH, Bethesda, MD). All experiments have been independently performed for three times.

### Flow cytometric analysis

HeLa and SiHa cells were transfected with NC miRNA, miR-519d inhibitor or shSmad7 oligo. Twenty-four hours after transfection, cells were exposed to 10 μM 5-fluorouracil (5-FU; Sigma, St Louis, MO) for an additional 24 h. Next, cells were harvested and flow cytometric analysis of cell apoptosis was carried out using an Annexin V-FITC/PI kit (BD PharMingen, San Diego, CA, USA) in accordance with the manufacturer’s instruments.

### MTT cell proliferation assay

MTT assay was performed using a protocol similarly to a previous report [17]. Briefly, HeLa and SiHa cells were plated into 96-well plates at a cell density of 2 × 10^3^ cells per well. At the indicated time points, cells were incubated with 20 μM MTT reagent in DMEM complete medium for 4 h. Thereafter, the medium was removed and 200 μl DMSO was added to incubate for an additional 20 min. The plate was read at 570 nm with a reference wavelength of 630 nm using an ELX Ultra Microplate Reader (Bio-tek, Winooski, VT, USA).

### Luciferase reporter assay

The 3′-UTR of Smad7 mRNA was subcloned into a Psi-CHECK2 luciferase reporter construct. The predicted miR-519d-binding site was mutated using overlap extension PCR to generate the Smad7mt-Luc construct. HeLa and SiHa cells were transfected with NC miRNA or miR-519d, together with Smad7wt-Luc or Smad7mt-Luc construct. Forty-eight hours after transfection, dual luciferase reporter assay was performed using Dual-luciferase reporter assay system (Promega, Madison, WI) in accordance with the manufacturer’s instruments.

### Statistical analysis

Statistical analyses were carried out using the SPSS 17.0 software package. Two-way analysis of variance (ANOVA), followed by a Student–Newman–Keuls post hoc test, was performed for the comparison between different groups of data. Student’s t test was applied when appropriate. P < 0.05 was considered statistically significant. All values were expressed as mean ± SE.

## Results

### Inhibition of miR-519d suppressed the proliferation and viability of cervical cancer cells

**Fig. 1:**
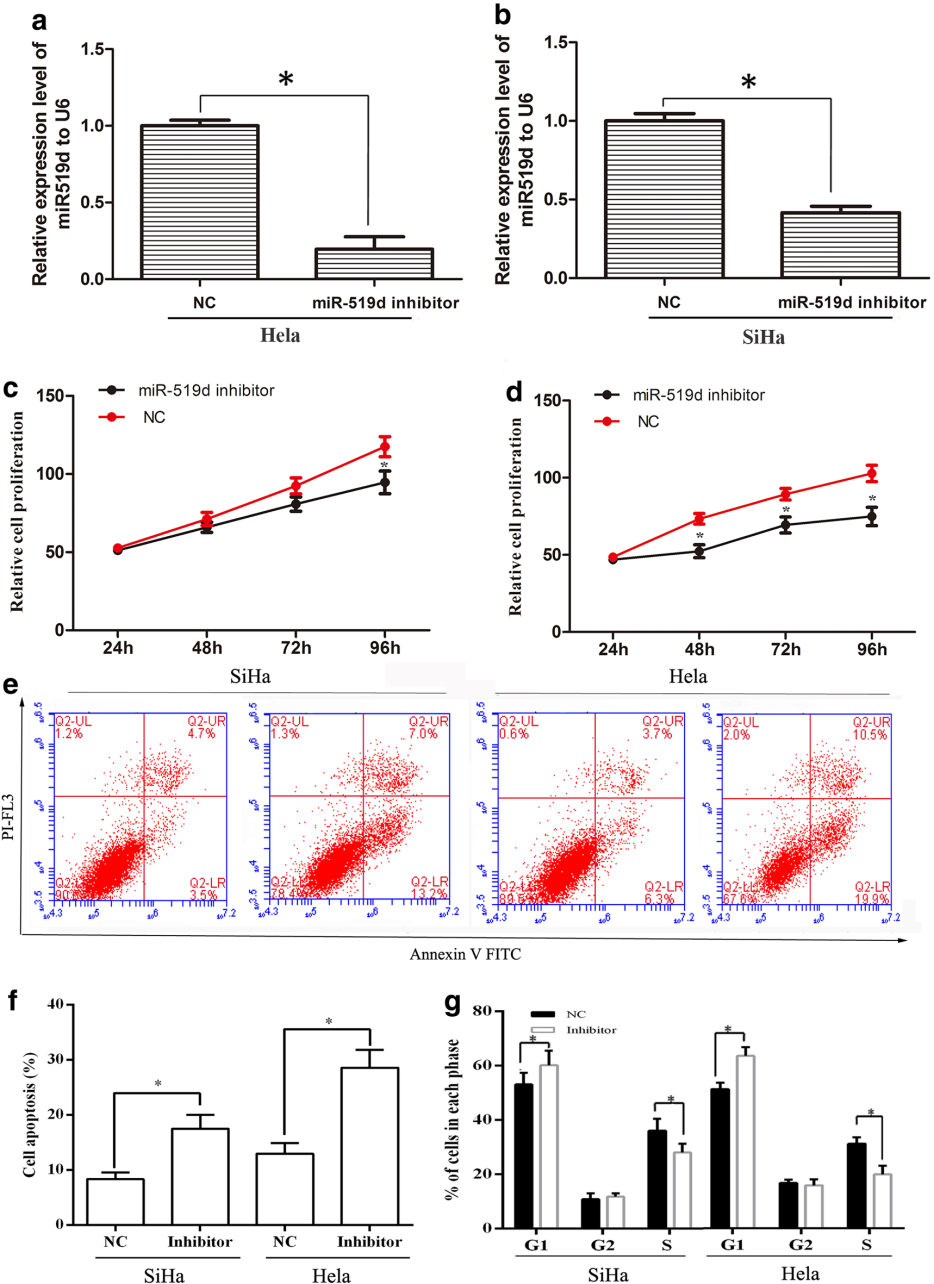
The impact of miR-519d inhibition on the proliferation and viability of cervical cancer cells. **a**, **b** The efficiency of miR-519d inhibitor has been detected by real-time PCR in Hela and SiHa cells (*P < 0.05). **c**, **d** HeLa and SiHa were subjected to MTT assay at different time periods (24, 48, 72 and 96 h) following transfection with NC miRNA or miR-519d inhibitor (*P < 0.05). **e** Annexin/PI apoptotic assay was performed to determine the proportion of apoptotic cells in HeLa and SiHa following transfection with NC miRNA or miR-519d inhibitor, along with 10 μM 5-FU treatment. **f** Statistical analysis of the proportion of apoptotic cells in the indicated groups (*P < 0.05). **g** Flow cytometrical analysis of cell cycle distribution of HeLa and SiHa cells following transfection with NC miRNA or miR-519d inhibitor (*P < 0.05)

In order to clarify the involvement of miR-519d in cervical cancer development and progression, miR-519d inhibitor was employed to investigate the impact of miR-519d inhibition on the physiology of cervical cancer cells. After transfection with miR-519d inhibitor, the cellular level of miR-519d was first determined using Real-time PCR analysis, the results showed that miR-519d expression level exhibited a significant decrease after transfection with miR-519d inhibitor (Fig. 1a, b). And then, the impact of miR-519d inhibitor on cell proliferation and viability was analyzed. As shown in Fig. 1c and d, transfected with miR-519d inhibitor significantly attenuated the proliferation of HeLa and SiHa cervical cancer cells. Furthermore, HeLa and SiHa cells were transfected with NC miRNA or miR-519d inhibitor, and then exposed to 10 μM 5-fluorouracil (5-FU) for 24 h. The cells were subjected to Annexin/PI apoptotic analysis, which showed that suppression of miR-519d distinctively augmented the proportion of apoptotic cells both in HeLa and SiHa cells (Fig. 1e, f). Moreover, flow cytometrical analysis indicated that transfection of miR-519d inhibitor augmented the proportion of cells in G1 phase, whereas reduced cells in S phase (Fig. 1g). These findings indicated that miR-519d might promote the proliferation and chemoresistance of cervical cancer cells.

### Inhibition of miR-519d attenuated the migration and invasion of cervical cancer cells

**Fig. 2:**
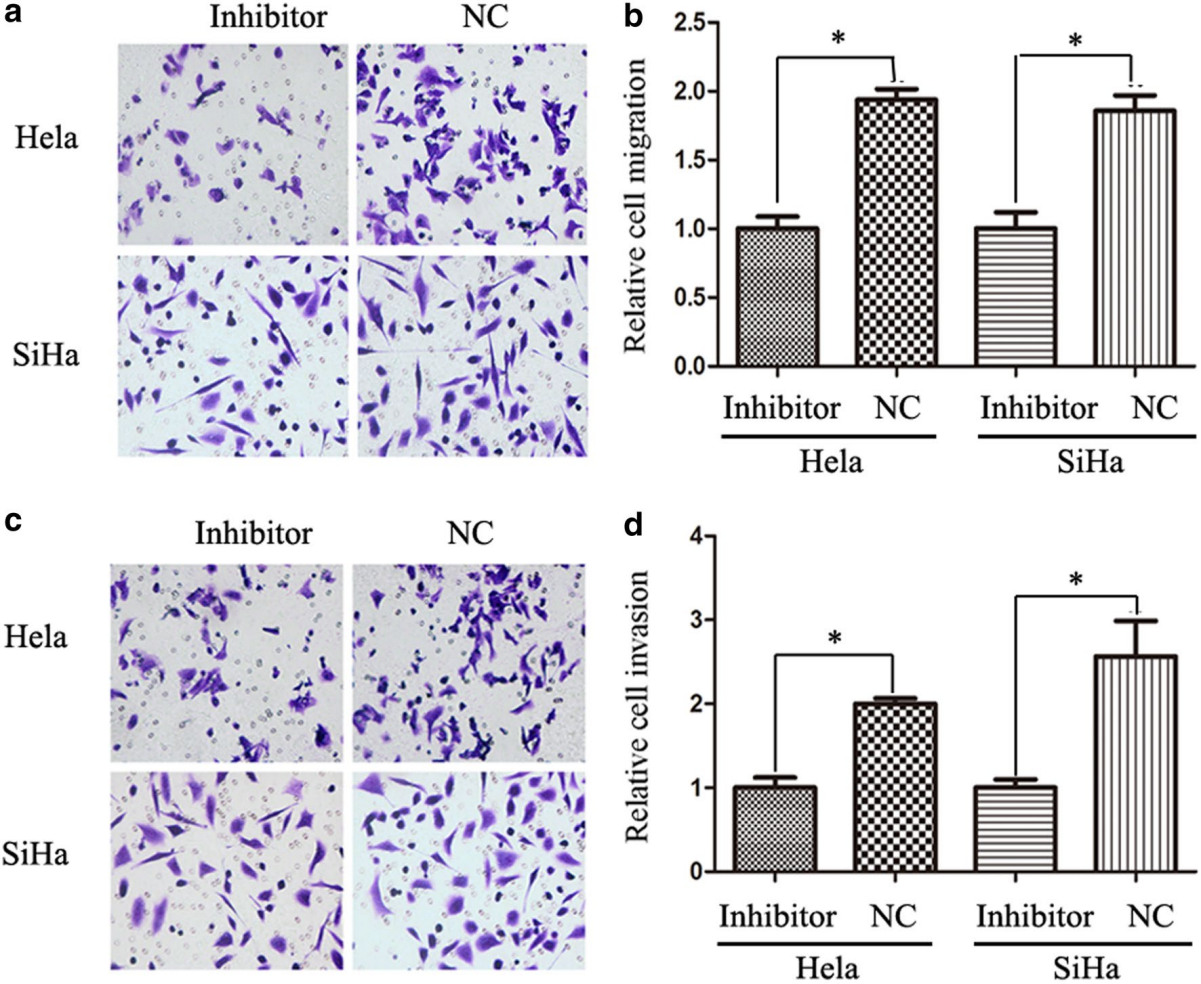
Transfection of miR-519d inhibitor restrained the migration and invasion of HeLa and SiHa cervical cancer cells. **a** Determining the impact of miR-519d inhibitor on the migration of HeLa and SiHa cells using transwell assay. The representative images showed that miR-519d inhibitor significantly attenuated the migration of HeLa and SiHa cells. **b** Quantitative analysis of the number of migrated cells in NC miRNA and miR-519d inhibitor groups (*P < 0.05). **c** Matrigel transwell analysis of the influence of miR-519d on the invasion of HeLa and SiHa cells. The representative images showed that miR-519d inhibitor significantly reduced the invasion of HeLa and SiHa cells. **d** Quantitative analysis of the number of cells invading through matrigel in NC miRNA and miR-519d inhibitor groups (*P < 0.05)

Next, the migration of HeLa and SiHa cells was analyzed using transwell assay. Transfection of miR-519d inhibitor markedly impaired the migrating capacities of both HeLa and SiHa cells (Fig. 2a). The number of migrated cervical cancer cells declined significantly following miR-519d inhibition (Fig. 2b). Moreover, the influence of miR-519d on the invasion of cervical cancer was evaluated using matrigel transwell assay. As predicted, transfection of miR-519d inhibitor reduced the number of cells invading through matrigel both in HeLa and SiHa cells (Fig. 2c, d).

### Smad7 is a novel target gene of miR-519d in cervical cancer cells

**Fig. 3:**
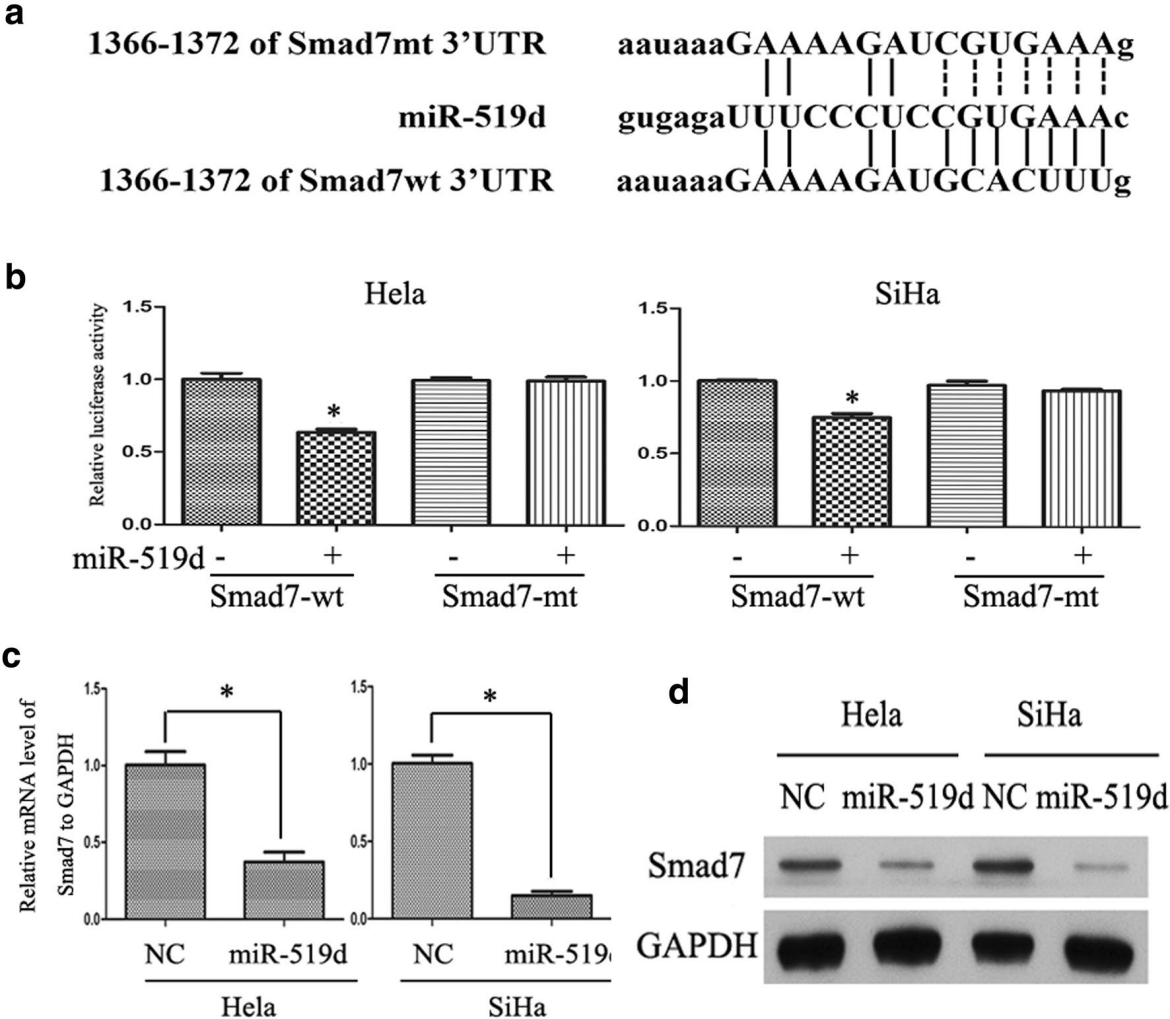
Smad7 is a novel target gene of miR-519d in cervical cancer. **a** Potential binding pattern of miR-519d to the 3′-UTR of Smad7 mRNA and the construct information of mutant Smad7 3′-UTR. **b** Firefly luciferase reporter assay through co-transfecting Smad7wt-Luc or Smad7mt-Luc with a Renilla luciferase control plasmid (pRL-TK), along with NC miRNA or miR-519d mimics. Shown were relative luciferase acitivities normalized to NC miRNA group (*P < 0.05). **c** Quantitative RT-PCR analysis of Smad7 mRNA in cervical cancer cells after transfection with NC miRNA or miR-519d mimics (*P < 0.05). **d** Western blot analysis revealed that the protein level of Smad7 was markedly reduced following miR-519d overexpression in HeLa and SiHa cells

Because miRNAs mainly modulate protein expression through direct RNA binding and decay, we were interested in the potential target genes involved in the tumorigenic role of miR-519d in cervical cancer. Using four independent miRNA target prediction softwares (miRanda, miRDB, miRWalk RNA22 and Targetscan), we identified that Smad7, a negative regulator of TGF-β signaling, as a novel candidate target gene of miR-519d. Next, we determined whether miR-519d might mediate the decay of Smad7 mRNA via direct association with mRNA 3′-UTR. To this end, the 3′-UTR of Smad7 mRNA was subcloned into Psi-CHECK2 luciferase reporter construct (designated as Smad7wt-Luc) (Fig. 3a). In addition, a mutant form of Smad7wt-Luc construct was generated through mutating the binding site of miR-519d (designated as Smad7mt-Luc) (Fig. 3a). Thereafter, the Smad7wt-Luc or Smad7mt-Luc construct was co-transfected with control miRNA or miR-519d into HeLa and SiHa cells. Forty-eight hours after transfection, the luciferase activity was assessed. As shown in Fig. 3b, miR-519d remarkably down-regulated the activity of Smad7wt-Luc both in HeLa and SiHa cells. While the activity of Smad7mt-Luc both in HeLa and SiHa cells were unchanged (Fig. 3b). Then, overexpression of miR-519d significantly attenuated the expression of Smad7 mRNA both in HeLa and SiHa cells (Fig. 3c). Likewise, the protein level of Smad7 also declined following miR-519d overexpression (Fig. 3d). These findings proved that the predicted binding site of Smad7 mRNA 3′-UTR played an essential role in miR-519d-modulated Smad7 expression, implicating that Smad7 is a direct target of miR-519d in cervical cancer.

### Interference of Smad7 restored miR-519d-mediated cervical cancer invasion and viability

**Fig. 4:**
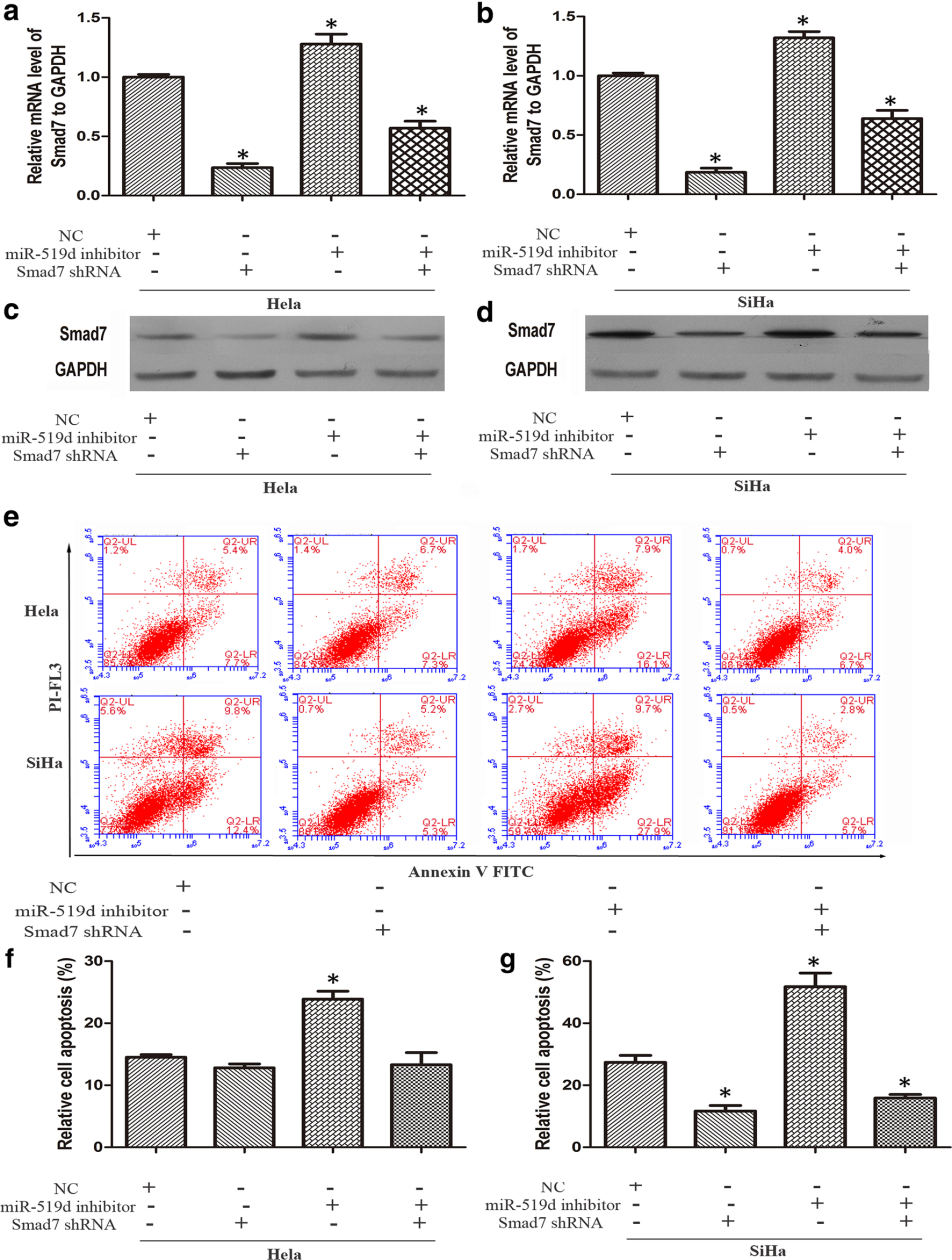
The effect of Smad7 depletion on miR-519d-mediated cervical cancer chemoresistance. **a**, **b** Real-time PCR analysis of Smad7 mRNA expression was performed after transfection of miR-519d inhibitor and Smad7 shRNA independently or simultaneously, the results were normalized to NC group. GAPDH was used as internal control (*P < 0.05). **c**, **d** Western blot analysis was performed to verify the protein expression level of Smad7 in the indicated groups. **e** After cell transfection and 5-FU treatment, Annexin V/PI analysis was performed to determine cell apoptosis in the indicated groups. **f**, **g** Statistical analysis of the proportion of apoptotic cells compared with NC group (*P < 0.05)

In the next step, we analyzed the involvement of Smad7 in the tumor-promoting role of miR-519d in cervical cancer. To this end, the Smad7-targeting shRNA oligo were employed to deplete endogenous Smad7 in cervical cancer cells. NC, miR-519d inhibitor and Smad7 shRNA oligo were transfected independently or simultaneously into cervical cancer cells. Real-time PCR analysis revealed that Smad7 shRNA oligo transfection group showed a significance reduced level of Smad7 mRNA compared with NC group. While the miR-519d inhibitor transfection group exhibited the highest expression level of Smad7 mRNA. And the Smad7 mRNA expression level of co-transfection group was below than NC group but higher than Smad7 shRNA oligo transfection group (Fig. 4a, b). In keeping with the real-time PCR results, western blot analysis displayed a similar varying tendency (Fig. 4c, d). The impact of Smad7 depletion on miR-519d-mediated cell viability was investigated. Then, we found that HeLa and SiHa cells exhibited distinct responses to Smad7, with regard to miR-519d-mediated anti-apoptotic effect. Whereas depletion of Smad7 restored the apoptotic response in miR-519d-inhibiting SiHa cells, HeLa cells did not display any difference in apoptotic rate in response to Smad7 interference (Fig. 4e–g). These findings inferred that other target genes probably played a regulatory role in miR-519d-triggered anti-apoptotic effect in HeLa cells.

**Fig. 5:**
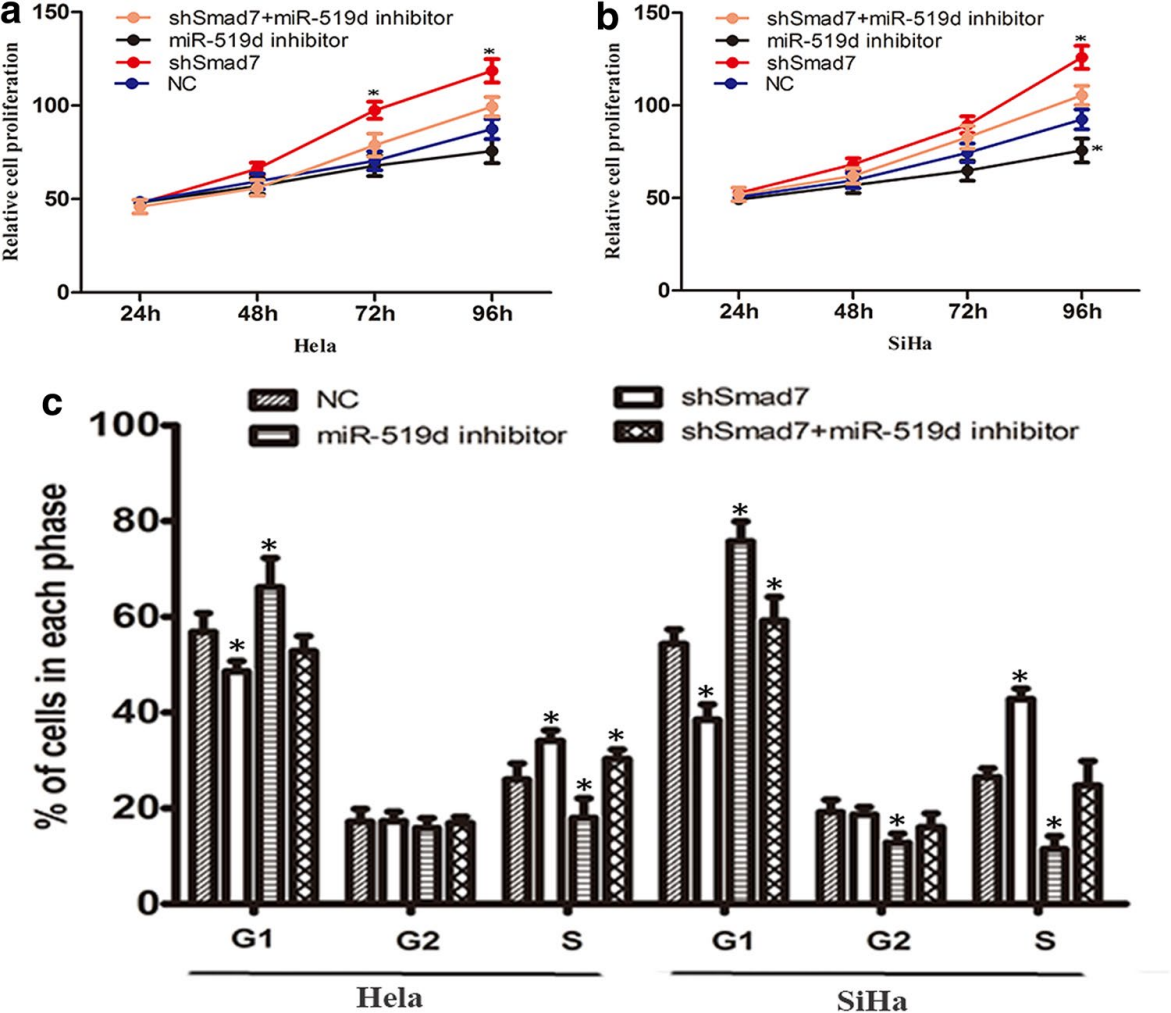
Depletion of Smad7 restored the cell proliferation and the cell cycle is disrupted in cervical cancer cells under miR-519d inhibition. **a**, **b** The proliferation rate of HeLa and SiHa cells were detected through MTT assay at different time periods (24, 48, 72 and 96 h) after transfection of miR-519d inhibitor and Smad7 shRNA independently or simultaneously compared with NC group (*P < 0.05). c Flow cytometrical analysis of cell cycle distribution was conducted in the indicated groups (*P < 0.05)

**Fig. 6:**
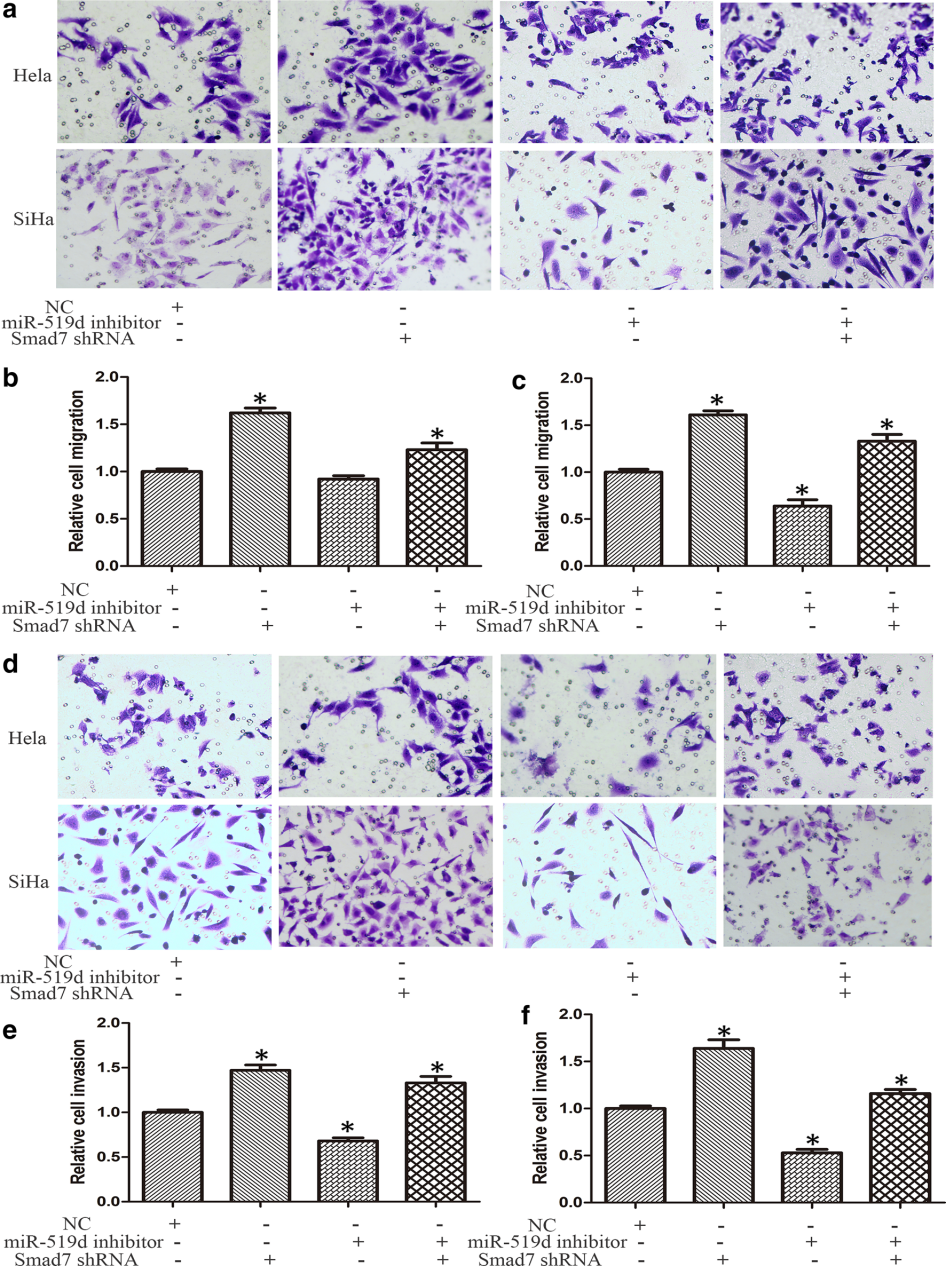
Depletion of Smad7 restored the migration and invasion of cervical cancer cells under miR-519d inhibition. **a** Transwell assay was performed to determine the migration capacity of HeLa and SiHa cells after transfection of miR-519d inhibitor and Smad7 shRNA independently or simultaneously compared with NC group. **b**, **c** Statistical analysis of the number of migrated cells in the indicated groups (*P < 0.05). **d** Matrigel transwell assay was performed to determine the invasion capacity of HeLa and SiHa cells after transfection of miR-519d inhibitor and Smad7 shRNA independently or simultaneously compared with NC group. **e**, **f** Quantitative analysis of the number of cells invading through matrigel in the indicated groups (*P < 0.05)

Furthermore, we analyzed the involvement of Smad7 in miR-519d-mediated cervical cancer migration and invasion. To this end, NC or miR-519d inhibitor plus with or without shSmad7 oligo were co-transfected into HeLa and SiHa cells. As shown in Fig. 5a and b, depletion of Smad7 by transfection of shSmad7 oligo significantly promote the proliferation of HeLa and SiHa cervical cancer cells compared with NC group. However, the proliferation of HeLa and SiHa cervical cancer cells were restored after co-transfection with miR-519d inhibitor. Furthermore, flow cytometrical analysis indicated that inhibiting Smad7 expression reduced the proportion of cells in G1 phase, whereas increased cells in S phase (Fig. 5c). And depletion of Smad7 induced an approximate 50 % upregulation of cell migration both in HeLa and SiHa cells (Fig. 6a–c). Likewise, matrigel transwell assay revealed that transfection of shSmad7 oligo resulted in comparable increase of cervical cancer cell invasion (Fig. 6d–f). Taken together, our findings conceivably validated that Smad7 potentially played an important role in miR-519d-mediated cervical cancer invasion and progression.

### MiR-519d was highly expressed in cervical cancer specimens

**Fig. 7:**
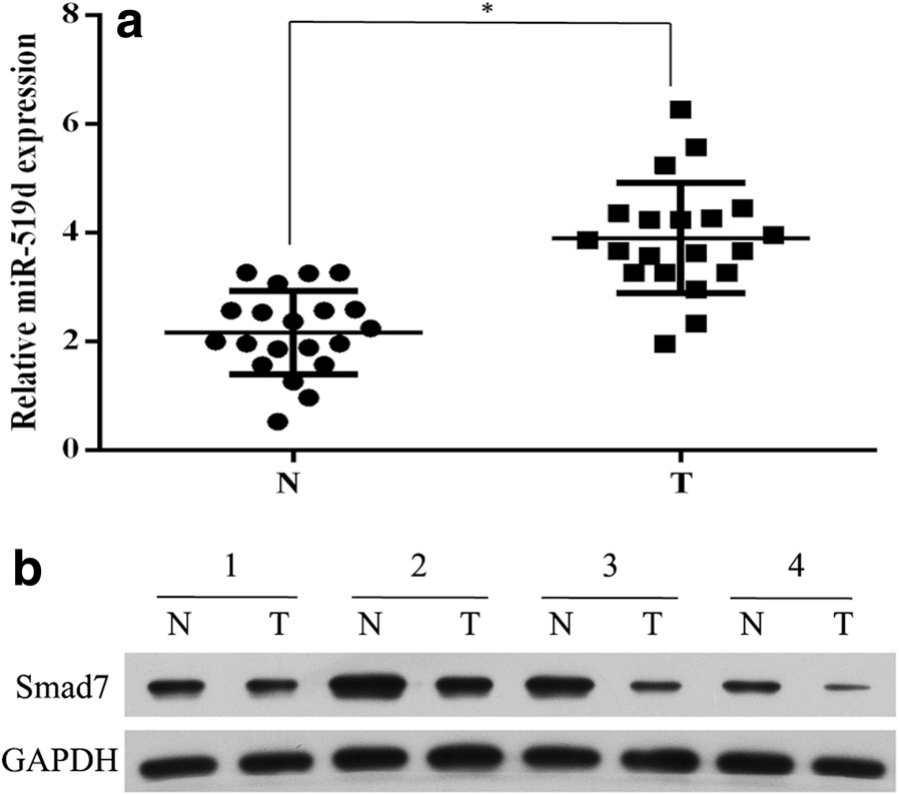
The expression pattern of miR-519d and Smad7 in cervical cancer and adjacent non-tumorous tissues. **a** The expression profile of miR-519d in non-tumorous cervical tissues (N) and cervical cancer samples (T) using real-time PCR analysis (*P < 0.05). **b** Western blot analysis of Smad7 expression in four paired non-tumorous cervical and cervical cancer tissues

MiR-519d was reportedly associated with the metastasis of osteosarcoma [18], and based on the above results, we confirmed that miR-519d significantly affected the progression and metastasis of cervical cancer through direct targeting Smad7. However, it was not clear whether miR-519d is highly expressed in cervical cancer tissues. In order to solve this problem, Real-time PCR was performed to check the differential expression of miR-519d between cervical cancer tissues and adjacent normal tissues. The results was shown in Fig. 7a, miR-519d is evidently upregulated in cervical cancer specimens. In addition, the expression of Smad7, a potential target of miR-519d, was also examined between cervical cancer and adjacent non-tumorous tissues, which found that Smad7 was downregulated in cervical cancer samples, compared with non-tumorous tissues (Fig. 7b). This piece of data verified that upregulated expression of miR-519d may contribute to the progression of cervical cancer.

## Discussion

Increasing evidence indicated that miRNAs played an integral roles in cervical tumorigenesis. Through screening tissue miRNA expression, a variety of miRNAs have been reported to be critically implicated in cervical cancer development and progression [19]. Smad7 is a critical inhibitor of TGF-β signaling pathway, which was reported to be expressed in tumor cells of numerous cancer types, including cervical cancer [20, 21]. In the present study, we for the first time showed that miR-519d was obviously upregulated in cervical cancer samples. Moreover, we found that miR-519d promoted the migration and invasion of cervical cancer cells. Additionally, miR-519d exerted an inhibitory role in the apoptosis of cervical cancer cells. Finally, we identified that Smad7 was a novel miR-519d target gene, and played a crucial role in miR-519d mediated tumor-facilitating effect in cervical cancer. These results uncovered a novel mechanism through which miR-519d promoted the viability and invasion of cervical cancer cells via suppressing Smad7 expression.

The importance of miRNAs in human tumor development has attracted significant attention in recent years. MiR-519d is located at chromosome 19 cluster, and was initially reported to target Ki-67, a proliferation marker protein, and suppress the in vitro growth of hepatocellular carcinoma cells [22]. However, later studies implicated that miR-519d also targeted several tumor suppressors, including CDKN1A/p21, PTEN and TIMP2, and thus potentially exerted oncogenic property in hepatocellular carcinoma development [15]. Indeed, miR-519d has been reportedly overexpressed in assorted human cancers, such as hepatocellular carcinoma, breast cancer, gastric cancer and clear cell renal cell carcinoma et al. [15, 23–26]. Accordingly, overexpression of miR-519d facilitated the proliferation, invasion and attenuated the apoptosis of cancer cells. However, notably, miR-519d exhibited remarkable downregulation in ovarian cancer [27]. In this respect, the biological significance of miR-519d promote cervical cancer development and progression remains virtually unknown. Our findings implicated that upregulated expression of miR-519d might contribute to cervical tumorigenesis via targeting Smad7, while depletion of Smad7 abrogated the influence of miR-519d on cervical cancer migration and invasion. Therefore, the precise role of miR-519d may vary between different cancer types, which may be attributed to different major targets in these cancers.

Metastasis represents the single most important prognostic factor predicting survival in cervical cancer [28]. However, the molecular mechanism underlying cervical cancer metastasis remains poorly understood. Several lines of studies indicated that TGF-β/Smads signaling played a vital importance in facilitating the metastasis of cervical cancer [4, 29, 30]. Therefore, it is important to clarify the mechanism underlying TGF-β hyperactivation in mCC. Serving as a potent inhibitor of TGF-β signaling, Smad7 is also a direct target of TGF-β/Smads pathway [31]. Thus, TGF-β-directed Smad7 expression functions as a negative feedback loop to prevent uncontrolled activation of TGF-β signaling. Low expression of Smad7 was associated with enhanced metastasis and poor prognosis in pancreatic cancer [32]. Consistent with these data, stable expression of Smad7 impairs tumor metastasis in vivo [6, 7]. However, the expression pattern and pathological significance of Smad7 in cervical cancer remains to be clarified. Interestingly, recent reports indicated that several miRNAs induced tumor metastasis through targeting Smad7 [33–36]. These findings suggested that miRNAs played an integral role in the regulation of Smad7 expression in tumor cells. Coinciding with this hypothesis, we found that miR-519d directly targeted Smad7 in cervical cancer cells. Suppression of miR-519d augmented Smad7 expression, leading to attenuated capacity of cervical cancer invasion. In addition to cell migration and invasion, our findings inferred that miR-519d regulated the apoptosis of cervical cancer cells. In this regard, we proposed that miR-519d might target other potential genes to regulate cervical cancer apoptosis, because transfection of shSmad7 oligo did not affect miR-519d’s anti-apoptotic function in SiHa cells. The detailed involvement of miR-519d in TGF-β signaling transduction and related metastatic pathways would be the subject of our future investigation.

In summary, our current study showed that miR-519d-mediated downregulation of Smad7 might contribute to cervical cancer invasion and metastasis. Using cervical cancer cell lines and specimens, we validated that miR-519d was associated with Smad7 downregulation and tumor metastasis in cervical cancer. Our findings implicated that targeting miR-519d expression might be a valuable approach to prevent the metastasis and chemoresistance of cervical cancer.

## Clinical practice points

Recent investigations indicated that cervical cancer development and progression involved dysregulated expression of various microRNAs. In this study, we for the first time identified that miR-519d, a miRNA that has been implicated in liver, breast and gastric carcinogenesis, was significantly upregulated in cervical cancer specimens, compared with non-tumorous cervical tissues. Additionally, we found that miR-519d directly targeted the mRNA of Smad7 to mediate the decay of Smad7 mRNA, thus facilitating the metastasis of cervical cancer. Using cervical cancer cell cultures, we showed that inhibition of miR-519d markedly impaired the migration and invasion of cervical cancer cells in vitro. Our findings imply that miR-519d may serve as a valuable indicator of cervical cancer metastasis. Furthermore, our results support the notion that miRNAs-based target therapy may be of potential clinical merit in cervical cancer management.
